# The complete mitochondrial genome of *Cichla ocellaris*

**DOI:** 10.1080/23802359.2017.1372713

**Published:** 2017-09-04

**Authors:** Minghui Lin, Xidong Mu, Xi Yan

**Affiliations:** a Pearl River Fisheries Research Institute, Chinese Academy of Fishery Sciences, Guangzhou, China;; b Key Laboratory of Freshwater Animal Breeding, Ministry of Agriculture, Huazhong Agricultural University, Wuhan, China

**Keywords:** Mitochondrial genome, Cichlaidae, *Cichlao cellaris*

## Abstract

In this study, the complete mitochondrial (mt) genome of *Cichlao cellaris* was determined. The mt genome has a length of 16,526 bp and encodes 13 protein-coding genes (PCGs), 22 transfer RNA genes, two ribosomal RNA genes, and one AT-rich non-coding region (D-loop). The gene arrangement was similar to those of typical fishes. The total base composition of the mt genome was 25.1% T, 30.8% C, 29.3% A, and 14.8% G. Of the 13 PCGs, 12 genes start with an ATG codon, except COX1 is with GTG. And among these 13 genes, seven (ND1, COX1, ATP8, ND4L, ND5, ND6, and Cytb) used TAA or TAG as the termination codon, whereas six (ND2, COX2, ATP6, COX3, ND3, and ND4) have incomplete stop codon T. Its control region is atypical in being short at 850 bp. This mt genome sequence data will be useful for phylogenetic and systematic analyses within the family Cichlaidae.

Mitochondrial (mt) genome sequences are widely used for comparative and evolutionary genomics, molecular evolution, phylogeny, population genetics, and species identification, due to its haploid nature, maternal inheritance and rapid evolutionary rate (Hulsey et al. 2007; Saitoh et al. 2011). Until now, several studies have reported the complete mt genome of the family Cichlaidae, such as *Oreochromis niloticus* (GenBank: GU370126), *Tropheus duboisi* (GenBank: AP006015), and *Astatotilapia calliptera* (GenBank: JN628855), and have resolved phylogenetic relationships among diverse groups of the family Cichlaidae (Mabuchi et al. 2007). The specimen of *Cichlao cellaris* was collected from the Huadiwan ornamental base, Guangzhou City, Guangdong Province, China. Genomic DNA extracted from fin clip tissue was stored in Pearl River Fisheries Research Institute. The complete mt genome of *C. ocellaris* was a closed-circular molecule of 16,526 bp in length with the typical gene content as other known Cichlaidae species: 13 protein-coding genes (PCGs), 22 transfer RNA genes (tRNA), two ribosomal RNA genes (rDNA), and one AT-rich non-coding region (D-loop). All genes were similar in length and identical in the gene arrangement to other Cichlaidae. The total base composition of *C. ocellaris* mt genome was 25.1% T, 30.8% C, 29.3% A, and 14.8% G, respectively. Of the 37 genes, 28 (two rRNAs, 14 tRNAs, and 12 PCGs) were encoded on the H-strand and nine (eight tRNAs and one PCGs) were encoded on the L-strand.

The encoding 13 PCGs was 11,431 bp in total length, with an average A + T content of 55.1%. Among the 13 PCGs, the highest A + T content was 56.3% in COX2, while the lowest was 48.5% in ND4L. Twelve of the 13 PCGs were coded on the heavy (H) strand, while ND6 was coded on the light (L) strand. Among the 13 PCGs identified, ranging in size from 183 bp (ATP8) to 1839 bp (ND5), 12 genes were inferred to use ATG as the start codon, whereas 1 gene (COX1) started with GTG. And among these genes, seven (ND1, COX1, ATP8, ND4L, ND5, ND6, and Cyt*b*) used TAA or TAG as the termination codon, whereas six (ND2, COX2, ATP6, COX3, ND3, and ND4) had incomplete stop codon T.

Two rDNA genes (12S and 16S rDNA) were encoded by the same H-strand with the 12S rDNA located between tRNA^Phe^ and tRNA^Val^, and 16S rDNA located between tRNA^Val^ and tRNA^Leu^. The 12S and 16S rDNA were 949 bp in length with 51.2% AT-content and 1693 bp in length with 55.2% AT-content, respectively. The typical set of 22 tRNA genes that ranged in size from 66 bp (tRNA^Cys^) to 74 bp (tRNA^Leu^ and tRNA^Lys^) was identified. The control region (D-loop, 850 bp) was located between tRNA^Pro^ and tRNA^Phe^ and has a high AT-content of 63.5% and was involved in the regulation of transcription and control of DNA replication (Boore 1999). Phylogenetic analysis using maximum likelihood method supported the monophyly of the family Cichlaidae and revealed that the genus *Cichla* and the genus *Retroculus* are clustered (Figure 1).

**Figure 1.**
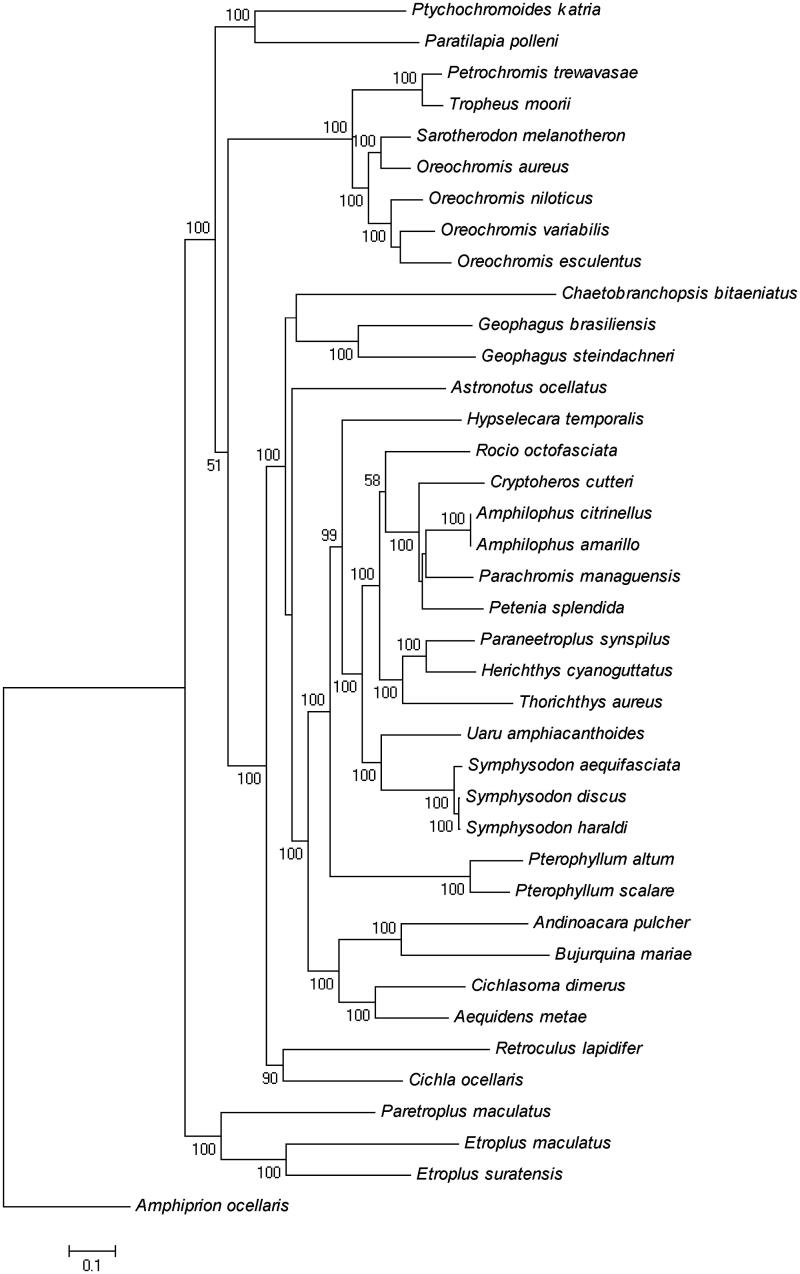
The phylogenetic relationships of the family Cichlaidae based on the nucleotide sequence of 13 protein-coding genes in the mitochondrial genome. The bootstrap support values with 1000 replicates are shown on the nodes. The *Amphiprion ocellaris* (AP006017) was used as outgroup.

## Nucleotide sequence accession number

The complete genome sequence of *C. cellaris* has been assigned GenBank accession number KU878410.
